# Network-Based Analysis Reveals Association of *FOXE1* Gene Polymorphisms in Thyroid Cancer Patients; A Case-Control Study in Southeast of Iran

**DOI:** 10.31557/APJCP.2020.21.9.2771

**Published:** 2020-09

**Authors:** Ahmad Mehrazin, Hossein Safarpour, Seyedeh Tahmineh Davoudi, Negin Parsamanesh, Farhad Saeedi, Ebrahim Miri-Moghaddam

**Affiliations:** 1 *Clinical Immunology Research Center, Faculty of Medicine, Zahedan University of Medical Sciences, Zahedan, Iran. *; 2 *Cellular and Molecular Research Center, Birjand University of Medical Sciences (BUMS), Birjand, Iran. *; 3 *Department of Biology, University of Sistan and Baluchestan, Zahedan, Iran. *; 4 *Student Research Committee and Dep. of Molecular Medicine, School of Medicine, (BUMS), Birjand, Iran. *; 5 *Student Research Committee, School of Medicine, (BUMS), Birjand, Iran. *; 6 *Cardiovascular Diseases Research Center and Department of Molecular Medicine, School of Medicine, Birjand University of Medical Sciences, Birjand, Iran. *

**Keywords:** Systems biology, WGCNA, thyroid cancer, FOXE1, polymorphism, genotype

## Abstract

Thyroid cancer (TC) is the mainly frequent endocrine cancer by different incidence rate in worldwide. However, early prediction of this cancer is still challenging due to the unclear pathogenicity. In this study with the aid of systems biology approach, performed a holistic study on GSE65144 dataset containing anaplastic thyroid carcinoma tissues. Co-expression network analysis by WGCNA suggested that highly preserved turquoise module with 1,480 genes was significantly correlated to TC. Most of the top 54 hub-genes of this module are functionality correlated to thyroid hormone generation (GO:0006590). Of these 54 hub-genes, *FOXE1* has been reported previously to contain mutation asosiated to TC and chosen for experimental validation step. To this end, we conducted a case-control study including 81 TC patients and 165 controls individuals to evaluate the effects of *FOXE1* functional polymorphisms (*rs1867277*) on the development of TC in Sistan and Balouchestan province of Iran. The polymorphisms of *FOXE1* gene (*rs1867277*) assessed by tetra-ARMS PCR technique. Homozygous (GG) and (AA) variant of* rs1867277 *polymorphism were detected in 26 (32.1%) and 15 (18.5 %) of TC patients, and 66 (40.0%), and 15 (9.1%) in controls, respectively (p-value= 0.03, OR= 2.53). The A allele frequency was 70 (43.2%) in TC patients and 114 (34.5%) in controls (p-value= 0.06, OR= 1.44). Overall, our results suggested that *FOXE1* gene could be used as a prognostic marker in TC and also provides information related to FOXE1 functional polymorphisms (*rs1867277*) in Sistan and Balouchestan province of Iran.

## Introduction

Thyroid cancer (TC) is the most widespread endocrine neoplasm by accounts 1% of entire human malignancies (Kondo et al., 2006). TC has a ninth place in the ranking of malignancies responsible for more than 500,000 cases worldwide. The global TC prevalence is 11 per 100,000 in women, and it is 3 times more than men population. In addition, TC represents 1 case in 20 cancer patients in 2018 and has low mortality rates (Bray et al., 2018; Horner et al., 2009). According to differentiation of cancer cells, TC classified into three groups, including papillary TC (PTC) as the most common type by 80 to 85% incidence rate, follicular TC (FTC) by 5 to 10% prevalence and Hurthle cell cancer as a rare type of TC (Davies and Welch, 2006). It seems that TC is the result of low to moderate penetration of multiple genes interactions with transcriptional regulators and collaboration of environmental risk factors that modulate TC susceptibility (Goldgar et al., 1994). Lack of iodine intake, exposure to ionizing radiation and certain pollutants particularly during childhood defined as environmental factors associated with TC risk (He et al., 2015). However, these explain just a small part of the TC etiology, thus diverse molecular signaling pathways and cellular conformations involved in TC development (Sierra et al., 2016). 

Even though TC harbors several highly universal genetic alterations, some of which are unique to this cancer (Xing, 2005). It is still very challenging to predict this cancer due to the complex disease progression process and complicated molecular interactions involved in it. Network medicine is a new approach that focuses on the application of systems biology to holistically study the molecular complexity of a particular disease (Fiscon et al., 2018; Lee and Loscalzo, 2019). On the basis of a theory that genes with a similar pattern of expression can have similar functions or take part in specific pathways, weighted gene co-expression network analysis (WGCNA) is used to explain gene correlation patterns across microarray and RNA-seq data in order to obtain co-expressed gene networks related to various diseases (Chen et al., 2019; Guo et al., 2018; Huayan and Runhong, 2019; Wang et al., 2019). WGCNA constructs a gene co-expression network that captures transcript relationships as defined in the pattern of gene expression in which genes in the same module may have similar functionality or may be regulated by regulatory factors (Langfelder and Horvath, 2007).

In this analysis, WGCNA was used to classify the significant modules of the co-expressed gene networks associated with TC. Finally, a case-control study on TC tissue samples was conducted to approve WGCNA results as well as finding new prognosis markers for TC.

## Materials and Methods

*Data collection and preprocessing*

The microarray data were taken from *NCBI* Gene Expression Omnibus (GEO) database with accession number GSE65144. This dataset is based on the GPL570 platforms and contain a total of 25 samples, including 13 anaplastic thyroid carcinoma tissue and 12 normal thyroid tissue. The raw data were corrected and quantile-normalized with the affy package of R 3.4.1 in Bioconductor. The annotation file published by Affymetrix (Affymetrix Human Genome U133 Plus 2.0 Array) was applied to assign probes for related gene IDs and symbols. Data of non-convertible probe IDs were excluded. The average statistics of identification expression for each sample were then conducted. Because of their varied, gene symbols were filtered through all samples, only the genes in the top 4,000 were selected for ensuing analysis. 

*Co-expression modules Construction of TC*

Co-expression network for patient and control group gene expression data maintained through two matrixes using WGCNA package protocols. The first matrix was developed according to the Pearson test by transforming the gene expression profile into a matrix of similarity between pair genes, however, the second matrix named adjacency matrix evolved from similarity matrix conversion. The WGCNA package developed scale-free gene co-expression networks. When the value was set to 6, the adjacency matrix met the scale-free topology criteria. The topological overlap is a metric of gene-biological similarity based on the pairwise gene-co-expression correlation. Topological overlaps matrix (TOM) and TOM dissimilarity (diss TOM) were obtained through TOM similarity and adjacency matrix dissimilarity. Finally, a minimum module size of 30 genes per module and a cut height of 0.1 were described as clusters of extremely interconnected genes.

*Module-trait relationships*

To identify modules closely related to the clinical trait, the expression profiles of each module were identified using the module’s eigenegene (ME) and the association between the module and the trait was analyzed. The role of human genes in TC was evaluated using the gene significance (GS) value. The Module Membership (MM) has been established as the ME correlation and gene expression profile for each module. Therefore, they can be used to set up the network and determine the hub genes. Specifically, genes with both GS ≥0. 74 and MM ≥0. 86 are considered to be hub genes.

*Functional enrichment of hub-genes*

Functional enrichment analysis of hub-genes was performed using Gene Ontology (http://geneontology.org/) and Kyoto Encyclopedia of Genes and Genomes (KEGG) (https://www.genome.jp/kegg/) databases. Enriched ontological terms and pathways with the threshold of Benjamin-adjusted p-value<0.05 were selected and visualized using ClueGO in Cytoscape ver 3.7.2. 

*Hub-gene prioritization by genetic variant analysis*

In order to investigate the impact of genetic variation, we extracted TC evidence for single-nucleotide polymorphisms (SNPs) from the GWAS catalog (Welter et al., 2013) resulting in 24 SNPs. Further on, these TC associated SNPs were filtered based on the similarity of mapped gene list and the obtained hub-genes from interest module. The similar genes were considered for next experimental validation.

*Sample collection*

In this case-control study, 81 TC patients were referred to the Imam Ali and khatam hospitals of Zahedan, City in the southeast of Iran during 2016-2018 and 165 healthy individuals without any history of neoplastic conditions were enrolled as volunteers. All of TC patients were histologically confirmed by pathologists and referred to Mehrazin nuclear medical center. The case and healthy participants were gender, age and ethnic matched. Informed consents were obtained from all participants before the sample collection. This study was signed informed consent code from the ethics committee of Zahedan University of Medical Science (IR. ZAUMS. REC.1397.289).

*Genotyping assay*

Genomic DNA was extracted from peripheral blood leukocytes of case and control group by routine extraction procure. *FOXE1* gene single nucleotide polymorphisms (SNPs) were genotyped using tetra-ARMS PCR. The primer sequences and product size have listed in Table 2. FOXE1 amplification program included an initial denaturation step (95°C for 5 min) followed by 28 cycles, 95°C, 1min; 58°C, 30 s; 72°C, 30 s with a final extension 5 min at 72°C. The PCR products were detected on safe stained (Cinna Gen, Iran) 2 % agarose gel following UV light visualization.

*Statistical analysis*

All statistical analysis was done by SPSS 19.0 software package (SPSS Inc., Chicago, IL). Descriptive statistics were carried out for determining the distribution of diverse genotypes within the case and healthy subjects. The p-value < 0.05 was considered significant.

## Results

*Identification of WGCNA modules*

Preprocessing of data, including quantile normalization was performed to reduce the effects of technical noises. The plot of quantiles of expression levels across arrays is shown in Figure. S1. A total of 4,000 genes were included in WGCNA based on a variance of expression values. Three outliers were found by sample clustering in 25 samples and 22 samples were therefore included in the analysis (Figure S2). Subsequently, β=6 was identified as a soft-threshold power for construction of weighted co-expression network (Figure S3). As a result, the hierarchical clustering dendrogram described six modules represented in different color branches of the dendrogram (Figure S4). The number of genes for each module ranged from 144 (green) to 1480 (turquoise) (Table 1).

*Module-trait association analysis*

**Figure 1:**
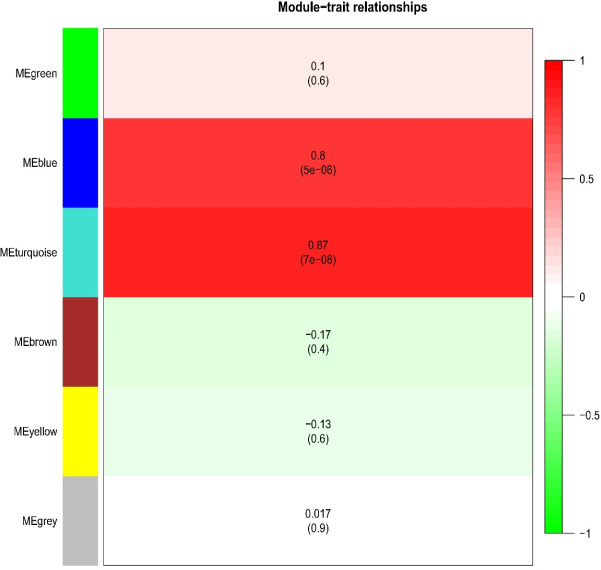
Module-Trait Relationship. Each row corresponds to a module eigengene and column corresponds to TC status. Numbers in each cell represent the corresponding correlation and p-value

**Figure 2:**
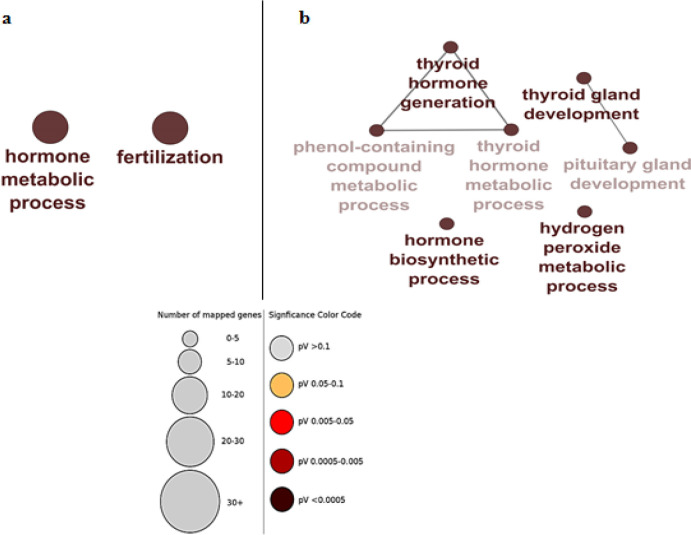
Processes and Pathways Identified Within the (a) turquoise module and hub-genes (b). Node size corresponds to the number of associated genes, and node color reflects the statistical significance. The darker the pathway node, the more statistically significant it is, with a gradient from red (p-value 0.05-0.005) to black (p-value < 0.0005)

Specific genes are determined for each module to evaluate the association of disease-presence modules in samples and module-module correlations the turquoise module as described was positively correlated with TC (r = 0.87, p-value = 7/00 E-08) (Figure 1). The most considerable pathways related to the turquoise module were visualized using the ClueGO tool. Hormone metabolic process and fertilization were the most important biological functions of the turquoise module (Figure 2A). 

*Identification of most significantly hub genes*

The correlation between MM and GS of the turquoise module has contributed to the discovery of hub-genes that are closely correlated with TC (Figure S5). The top 54 hub-genes with GS ≥0.74 and MM ≥0.86 are listed in Figure S6. In our study, we examined the Enrichr analysis of our hub genes and found that thyroid hormone generation (GO: 0006590) including FOXE1, IYD, and DUOX2 is the most important biological process. In addition, KEGG 2019 Human analysis found that thyroid hormone synthesis is the most important pathway, including TPO, IYD, DUOXA2, LRP2, DUOX2, and TSHR (Figure 2B). 

**Figure 3:**
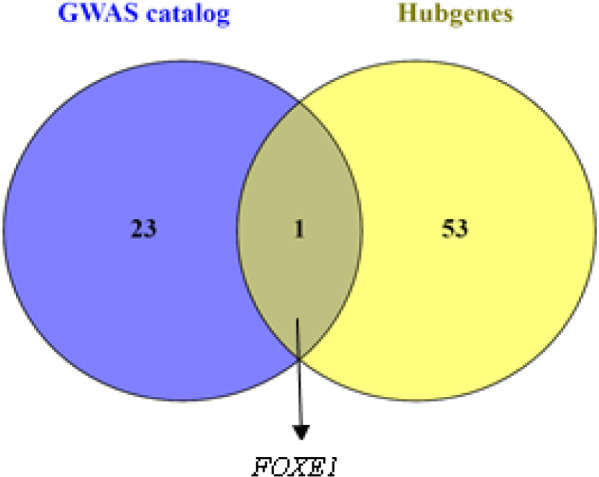
Similarity Analysis within Obtained Hub-Genes and GWAS Catalog. The result indicated that only *FOXE1* was common to both list

Furthermore, to find that which hub-gene SNPs were associated with TC pathogenicity, similarity analysis was conducted using Venny online program (https://bioinfogp.cnb.csic.es/tools/venny/) within obtained hub-genes and GWAS catalog containing 24 different SNPs for TC. The result indicated that only FOXE1 was common in both list. So, this gene was considered for next experimental validation (Figure 3). 

*Tissue validation for FOXE1 polymorphism (rs1867277)*

In the current study, *rs1867277* polymorphism of *FOXE1* gene was assessed in 81 TC patients and 165 healthy individuals of southeast of Iran. From overall 246 individuals, 90 (36.5%) and 156 (63.4%) were males and females respectively. There was not any considerable difference in male and female distribution in healthy individuals 58 (35.2%) and 107 (64.8%) and TC patients 32 (39.5%) and 49 (60.5%), respectively. The mean age of the patients and healthy individuals were 46 ± 11.4 and 42±7.2, respectively. The results indicated that in rs1867277, the frequency of homozygous AA was 18.5% and 9.1% of patients and the control, respectively (p-value = 0.03, OR= 2.53). Also A allele frequency was 43.2 % of patients and 34.5% in the healthy group (p-value = 0.06, OR= 1.44) (Table 3).

**Table 1:** Module Colors Characterization. The co-expression modules identified by WGCNA

Module color	#Genes	Correlation	p-value
Turquoise	1480	0.87	7.00E-08
Blue	812	0.8	5.00E-06
Green	144	0.1	0.6
Grey	549	0.017	0.9
Yellow	210	-0.13	0.6
Brown	805	-0.17	0.4

**Table 2:** Primers Sequencing and Amplicons Size were Used for *FOXE1* Genotyping

SNP	Primers sequence	Amplicon size (bp)
	F-outer: 5′-TAA ACT AGC GGG CAC CAC AGA CC-3′	Outers: 309
rs1867277	R-outer: 5′-AGA GCT CAG GGG ATC GTC GC-3′	Inner A:144
	F-inner: 5′-AGA GTC CAG TCC CGG GCG-3′	Inner G: 201
	R-inner: 5′-CAG CGG CGG TGG CCT AGT-3′	

**Table 3:** *FOXE1* Genotypic and Allelic Frequency in TC Patients (n=81) and Healthy (n=165) Objects

Gene	Accession number	Chromosome location	Genotype	Case N (%) N = 81	Control N (%) N = 165	p-value	OR
			GG	26 (32.1)	66 (40.0)	Ref:1	
			AG	40 (49.4)	84 (50.9)	0.52	1.2
*FOXE1*	rs1867277	9q22.33	AA	15 (18.5)	15 (9.1)	0.03*	2.53
			AG+AA	55 (67.9)	99 (60.0)	0.23	1.41
			Allele G	92 (56.8)	216 (65.5)	Ref:1	
			Allele A	70 (43.2)	114 (34.5)	0.06	1.44

*, Significant values in bold. FOXE1 genotypes (GG, wild type; AG, heterozygote; AA, mutant type).

## Discussion

TC is the multifactorial disease by 1% prevalence of the entire human cancers and known as the most frequent endocrine malignancy (Liyanarachchi et al., 2013; Nguyen et al., 2015). Genetic predisposition, radiation exposure and hormonal factors are the possible causes of TC etiology, but the exact reason is still poorly understood (Schmidbauer et al., 2017). Despite the recent development of diagnostic and therapeutic approaches, the prognosis of TC remains poorly understood (Saini et al., 2018). Nowadays, the main public health goal is to develop biomarkers and targeted therapy approaches for early diagnosis and treatment of TC, respectively (Abdullah et al., 2019). To this end, our study was conducted to identify biomarkers associated with progression of TC using WGCNA. In the present study, we have analyzed GSE65144 dataset containing 25 samples including 13 anaplastic thyroid carcinoma tissue and 12 normal thyroid tissue. Co-expression network analysis by WGCNA suggested that highly preserved turquoise module with 1,480 genes was significantly correlated to TC. The top 54 hub genes of this module were listed in Fig. S6, most of them are functionally correlated with thyroid hormone generation (GO: 0006590). Similarity analysis between obtaining hub-genes and GWAS catalog revealed that, from this hub-genes list, only FOXE1 has been reported previously to contain a mutation associated with TC. FOXE1 is a kind of thyroid transcription factors that play tumor suppressor role in the control of cell proliferation and tumor invasion in thyroid cells (Parlato et al., 2004). The cellular transcription factor 1 belongs to the basic helix-loop-helix group (Takahashi et al., 2010). The rs1867277 of* FOXE1* gene is an A/G single-nucleotide variation in the 5′ untranslated region on human chromosome 9 (Landa et al., 2009). It was connected to a gene modulation of the transcriptional regulators. Previously, it has been demonstrated significant association between rs1867277 variation and differentiated TC of the many different populations (Pereda et al., 2015). On this basis, *FOXE1* gene was considered for next experimental validation. Evidence indicated that *FOXE1* gene can enhance the susceptibility of sporadic TC in a Japanese and European population (Gudmundsson et al., 2009; Matsuse et al., 2011). To this end, we conducted a case-control study to evaluate the effects of *FOXE1* functional polymorphisms (*rs1867277*) on the development of TC in Sistan and Balouchestan province of Iran.

The result of our study showed a AA genotype frequency of 18% and 9% in case and control (p-value = 0.03, OR = 2.53). In onother studies, for example, Somuncu et al., (2015) it is shown that AA genotype of rs1867277 variation is considerably related to numerous histopathological parameters. In addition, AA genotype was notably linked with the classical variant that belongs to PTC. Kula et al., (2017)confirmed the positive association between rs1867277 and PTC predisposition in Polish population (p-value = 1×10^-6^, OR = 1.59) . In other study, Nikitski et al., (2017) indicated remarkable relation between FOXE1 coding region and PTC of functional SNPs rs1867277 in the Japanese and Belarusian populations by univariate analysis). Also, Landa et al., (2009) indicated that A allele of rs1867277 was related to TC risk. Furthermore, in another study, A allele reported significantly related to TC and they showed that *FOXE1* polymorphisms were associated with risk of PTC (Somuncu et al., 2015). Further, Kula et al., (2010) showed, A allele was probably associated with TC in the metastatic form. FOXE1 is necessary for proliferation and migration of thyroid cells during morphogenesis. Hence, clarified of FOXE1 risk variants and their functions can be fundamental in tumor transformation (Tomaz et al. 2012). On the other hand, FOXE1 as thyroid particular DNA binding could identify thyroperoxidase and thyroglobulin (Cuesta et al., 2007; Fernández et al., 2013). In this regard, Three cohort studies, confirmed the positive association in the rs1867277 and the *FOXE1* poly Ala tract polymorphism with TC in Portuguese, Australian and Spanish population (Bullock et al., 2012; Kallel et al., 2011; Tomaz et al., 2012). FOXE1 is essential for thyroid and pituitary gland formation and play in the key role in the regulatory function of transcription factors that involved in thyroid differentiation in the embryonic phase. 

In conclusion, via systems biology approach and co-expression network analysis, we identified a novel module involved in TC pathogenicity and discovered main hub-genes e.g. *FOXE1*. To confirm bioinformatic result, a case-control study was conducted on tissue samples to evaluate associations between* FOXE1* (*rs1867277*) polymorphisms with TC. The results showed a significant correlation among rs1867277 and TC pathogenicity, suggest that FOXE1 (rs1867277) SNP can be a potential predictor biomarker for TC diagnosis. Moreover, functional studies are required to confirm its role as a therapeutic target. We recommend detailed assessing these polymorphisms in larger populations of various geographical regions. 
